# Making human papillomavirus testing a public health priority in Ecuador

**DOI:** 10.3389/fpubh.2025.1535580

**Published:** 2025-05-27

**Authors:** Gustavo Galárraga, José Francisco López-Gil

**Affiliations:** ^1^School of Public Health, University of Minnesota, Minneapolis, MN, United States; ^2^One Health Research Group, Universidad de Las Américas, Quito, Ecuador

**Keywords:** cervical cancer, public health, pap smear, vaccination programs, screening policies, health equity, health disparities

## Abstract

Cervical cancer is a pressing public health issue in Ecuador, where it is the second most common cancer among women, with 1792 new cases and 939 deaths annually. Despite the availability of superior diagnostic methods like human papillomavirus deoxyribonucleic acid testing, the national strategy continues to prioritize Pap smears, limiting the effectiveness of early detection efforts. Disparities in screening access are evident, with only 54% of women aged 12–49 undergoing Pap smears, and coverage is even lower in marginalized populations. Learning from Peru’s National Cancer Law, Ecuador has the opportunity to adopt similar measures, ensuring free and equitable access to human papillomavirus (HPV) testing. By scaling up human papillomavirus screening, integrating vaccination programs, and addressing socioeconomic barriers, Ecuador can move closer to eliminating cervical cancer as a public health threat. The time to prioritize human papillomavirus testing is now.

## Introduction

Cervical cancer is one of the leading public health challenges globally and disproportionately affects women in low- and middle-income countries. According to Global Cancer Observatory (GLOBOCAN), cervical cancer ranks as the second most common cancer among women in Ecuador, with 1792 new cases and 939 deaths reported annually (1). This aligns with global estimates showing high burdens in low-resource settings, particularly in regions where screening and vaccination programs are limited (2, 3).

For decades, the Pap smear has been the primary method for cervical cancer screening in Ecuador, despite its known limitations. Studies have shown that human papillomavirus (HPV) deoxyribonucleic acid (DNA) testing has superior sensitivity and predictive power compared to Pap smears, making it the recommended method for early detection of cervical cancer (4–6). By detecting high-risk HPV genotypes, such as 16 and 18, or those with potential carcinogenic risk, including 26, 30, 34, 53, 66, 67, 69, 70, 73, and 82, it is essential to ensure proper testing and appropriate clinical management, as these should not be taken lightly (7). HPV testing offers a more accurate method of identifying precancerous lesions before they develop into invasive cancer (3, 8). Modeling analyses further suggest that transitioning to HPV-based screening programs in low- and middle-income countries could prevent millions of deaths and significantly reduce the burden of cervical cancer (6). In Ecuador studies have found a cost-effectiveness of HPV testing over pap smear in a 5-year period is $1,085.75 from the government’s perspective, and it results in savings of $5,926.90 from the society’s perspective (10).

*“I want to share my story anonymously to help others. When I tested positive for HPV (human papillomavirus), I was terrified. I thought I had cancer and blamed myself for contracting the virus. I had been doing annual Pap smears since I was 18, and every result had been normal. I followed the instructions, repeated the test every year, and moved on. However, I began experiencing a persistent discharge that lasted for an entire month. I thought it was normal—but it wasn’t. Something felt off. A friend, who knew about HPV, suggested I undergo DNA testing for HPV. Days later, the results came back….”*

Anonymous patient.

## The current reality in Ecuador

National data reveals significant disparities in cervical cancer prevention.

Despite the HPV test costing $75.80 per test and being offered for free in public clinics, data from the 2018 National Health and Nutrition Survey (ENSANUT) shows that only 54% of women aged 12–49 reported ever having undergone a Pap smear and no data available in HPV testing at a national scale (9–12). These numbers drop further among women with lower educational attainment, with just 49% of women with basic education and 52% of those with high school education accessing screening. Such disparities underscore the need for a more equitable and accessible approach to cervical cancer prevention.

In 2023, the Ministry of Public Health took a significant step by introducing HPV molecular testing at 36 national screening centers, targeting 40,000 women aged 30–65 years for genotypes 16 and 18 (13). While this initiative is commendable, it is far from sufficient to meet the needs of the population. HPV testing remains unavailable to many women in rural areas and those from lower socioeconomic backgrounds, perpetuating existing health inequities (12).

*“When I saw my results, all the high-risk genotypes, such as 16 and 18, were negative. But one stood out—genotype 34 was positive. The diagnosis led to painful treatments, including colposcopy and fulguration. The doctor informed me that the virus would remain in my body, but with regular monitoring and the right treatment, I could significantly lower my risk of developing cervical cancer. There was hope at last.”*

Anonymous patient.

## Lessons from regional policies

Ecuador can learn from the experiences of neighboring countries. In 2022, Peru enacted a National Cancer Law that mandates free HPV testing in all public health facilities (14). This policy has been transformative, ensuring that women, regardless of their socioeconomic status, have access to the most effective screening method. However, in Ecuador the only document that stands as a statement for cancer is the outdated document since 2022 of the “*Estrategia Nacional para la Atención Integral del Cáncer en el Ecuador*” that serves as a guideline for cancer but not a nation priority under legal normative (15). Adopting a similar framework in Ecuador could bridge the gaps in access and ensure that HPV testing becomes a universal right rather than a privilege.

Moreover, Ecuador has recently made progress in primary prevention. In May 2024, the Ministry of Public Health expanded its HPV vaccination program to include boys aged nine (16). This move aligns with global strategies that emphasize the integration of vaccination and HPV testing to achieve cervical cancer elimination (4, 5, 17). Comprehensive approaches that include vaccination, universal HPV screening, and timely treatment have been shown to accelerate the elimination of cervical cancer in low-resource settings (6).

## The path forward

HPV testing is not just a diagnostic tool—it is a gateway to health equity and a means to save lives. A comprehensive strategy to address cervical cancer in Ecuador should include:

1. *Scaling up HPV testing.* Expand the availability of HPV testing beyond the current pilot programs to include all public health facilities nationwide.
2. *Investing in education campaigns.* Raise awareness about HPV, its risks, and the importance of testing among women, men, and healthcare providers. Misconceptions about the virus and stigma associated with sexually transmitted infections must be addressed through targeted communication strategies (17).
3. *Reducing socioeconomic barriers.* Ensure that HPV testing and treatment are free or affordable, especially for women in rural and underserved communities (9).
4. *Integrating men into prevention efforts.* Include men in educational campaigns to promote shared responsibility in preventing HPV transmission (16).
5. *Leveraging technology.* Use digital tools to track and monitor HPV screening rates, ensuring data-driven decision-making and identifying gaps in service delivery.

As the WHO’s global strategy to eliminate cervical cancer highlights, combining universal vaccination, HPV testing, and access to treatment can make this disease a problem of the past (18). Ecuador has the tools to achieve this goal, but it requires urgent action to prioritize prevention, reduce inequalities, and ensure that no woman is left behind.

*“My message to other women is to learn about HPV risks, to discuss the issue openly with friends and partners. There are ways to prevent and treat it, but first, we must know the options available to us. I hope my story inspires others to seek the right tests and take control of their health.”*

Anonymous patient.

Ecuador has the tools to make cervical cancer a disease of the past. By prioritizing HPV testing, expanding vaccination programs, and addressing inequalities in access to care, we can move closer to achieving health equity and protecting future generations from this preventable disease. The time to act is now.

## Data Availability

The original contributions presented in the study are included in the article/supplementary material, further inquiries can be directed to the corresponding author.
